# Double Trouble: Guillain-Barré Syndrome (GBS) Presenting as Overlapping Miller Fisher Syndrome (MFS) and Pharyngeal-Cervical-Brachial (PCB) Variant

**DOI:** 10.7759/cureus.98367

**Published:** 2025-12-03

**Authors:** Amit Kumar, Sandeep Garg, Praveen Bharti, Bhvika Zutshi

**Affiliations:** 1 General Medicine, Maulana Azad Medical College, New Delhi, IND; 2 Medicine, Maulana Azad Medical College/Lok Nayak Hospital, New Delhi, IND; 3 Medicine, Maulana Azad Medical College, New Delhi, IND

**Keywords:** acute flaccid paralysis, anti-gq1b antibody, guillain-barré syndrome, miller fisher syndrome, pharyngeal-cervical-brachial variant

## Abstract

Guillain-Barré Syndrome (GBS) is the leading cause of acute flaccid paralysis in India following the decline of poliomyelitis. Classical GBS typically presents with ascending symmetrical weakness and areflexia, while atypical variants, such as Miller Fisher Syndrome (MFS) and the Pharyngeal-Cervical-Brachial (PCB) variant, show distinct clinical patterns that are often under-recognized, particularly in resource-limited settings. This report describes a rare case of a 45-year-old previously healthy woman who developed progressive ophthalmoplegia, bulbar weakness, neck flexor weakness, and proximal upper limb weakness, along with areflexia, following a mild upper respiratory tract infection. Sensory function and lower limb motor strength remained intact. The differential diagnoses included brainstem stroke, myasthenia gravis, and diphtheritic polyneuropathy. Cerebrospinal fluid analysis revealed albuminocytologic dissociation, and nerve conduction studies showed a demyelinating sensorimotor polyneuropathy predominantly affecting the upper limbs. The presence of anti-GQ1b and anti-GT1a IgG antibodies supported an immune-mediated process. A diagnosis of GBS with overlapping MFS and PCB variants was established. The patient received a five-day course of intravenous immunoglobulin along with supportive management. Her neurological function gradually improved, with complete recovery noted at the three-month follow-up. This case highlights the importance of early recognition of overlapping GBS variants, especially in patients with cranial nerve and upper limb involvement. Greater clinical awareness and improved access to ganglioside antibody testing may facilitate earlier diagnosis and better outcomes. The coexistence of MFS and PCB variants also reinforces the concept of GBS as a spectrum disorder with shared underlying mechanisms.

## Introduction

Guillain-Barré Syndrome (GBS) is an acute, immune-mediated polyradiculoneuropathy and is now the leading global cause of acute flaccid paralysis following the decline of poliomyelitis. In India, GBS has become a notable neurological emergency, with an estimated annual incidence of 1.5-2.7 per 100,000 population, though this is likely underestimated due to limited diagnostic access in rural regions [1,2]. Infectious triggers, such as *Campylobacter jejuni*, cytomegalovirus, Epstein-Barr virus, and *Mycoplasma pneumoniae, *commonly precede the illness and initiate an autoimmune response through molecular mimicry [3].

GBS usually presents with ascending symmetrical weakness, areflexia, and varying degrees of sensory or autonomic dysfunction. Typical variants include acute inflammatory demyelinating polyneuropathy (AIDP), acute motor axonal neuropathy (AMAN), and acute motor-sensory axonal neuropathy (AMSAN). Atypical variants, however, have been increasingly recognized, particularly in Asia. Among these, Miller Fisher Syndrome (MFS) and the Pharyngeal-Cervical-Brachial (PCB) variant are rare but clinically important subtypes [4].

MFS, first described in 1956, is defined by the triad of ophthalmoplegia, ataxia, and areflexia, and accounts for approximately 1%-5% of global GBS cases. It is more commonly observed in East Asia and is strongly associated with anti-GQ1b IgG antibodies, present in more than 85% of patients. This supports an autoimmune process targeting cranial nerves and neuromuscular junctions [5].

The PCB variant, described by Ropper in 1986, manifests with acute oropharyngeal, cervical, and upper limb weakness, with minimal or no lower limb involvement. Because of its presentation, it can mimic disorders such as myasthenia gravis or brainstem stroke, making diagnosis challenging. Anti-GT1a or anti-GQ1b antibodies are frequently detected in PCB cases, suggesting shared immunopathological mechanisms with other GBS variants [6].

Cases demonstrating overlapping features of MFS and PCB variants are uncommon but clinically significant. These presentations blur strict diagnostic categories and reinforce the concept of GBS as a spectrum rather than a collection of isolated syndromes. Such overlaps are especially vulnerable to misdiagnosis or delayed recognition in resource-limited settings where ganglioside antibody testing is unavailable or prohibitively expensive.

## Case presentation

A 45-year-old previously healthy woman presented to the emergency department with a 12-day history of progressive diplopia, drooping of the eyelids, dysarthria, and dysphagia, accompanied by neck flexor weakness and an inability to raise both arms above shoulder level. She reported no fever, limb paresthesia, trauma, or bowel or bladder disturbances. One week earlier, she had experienced a mild upper respiratory tract infection, characterized by sore throat and rhinorrhea, which resolved spontaneously.

**Figure 1:**
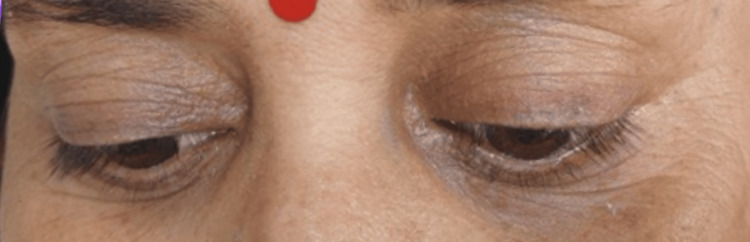
Bilateral ptosis in the patient.

On examination, she was alert and oriented, with no signs of encephalopathy or meningeal irritation. Cranial nerve evaluation revealed bilateral external ophthalmoplegia with complete restriction of eye movements in all directions, along with partial bilateral ptosis (Figure 1). Assessment of the seventh cranial nerve showed bilateral lower motor neuron facial weakness. There was no gag reflex or palatal elevation, and her speech was dysarthric with a nasal quality.

On motor examination, neck flexor strength was reduced to Medical Research Council (MRC) grade 3/5, and proximal upper limb weakness was noted, with deltoids and biceps graded at 2/5 bilaterally. Distal upper limb strength and all lower limb muscle power were normal. Deep tendon reflexes were absent throughout. Sensation, coordination, and gait were normal in all limbs; however, the patient was unable to sit or walk independently due to truncal instability and neck weakness. The differential diagnoses considered included brainstem stroke, myasthenia gravis, botulism, diphtheritic polyneuropathy, and GBS variants.

**Figure 2:**
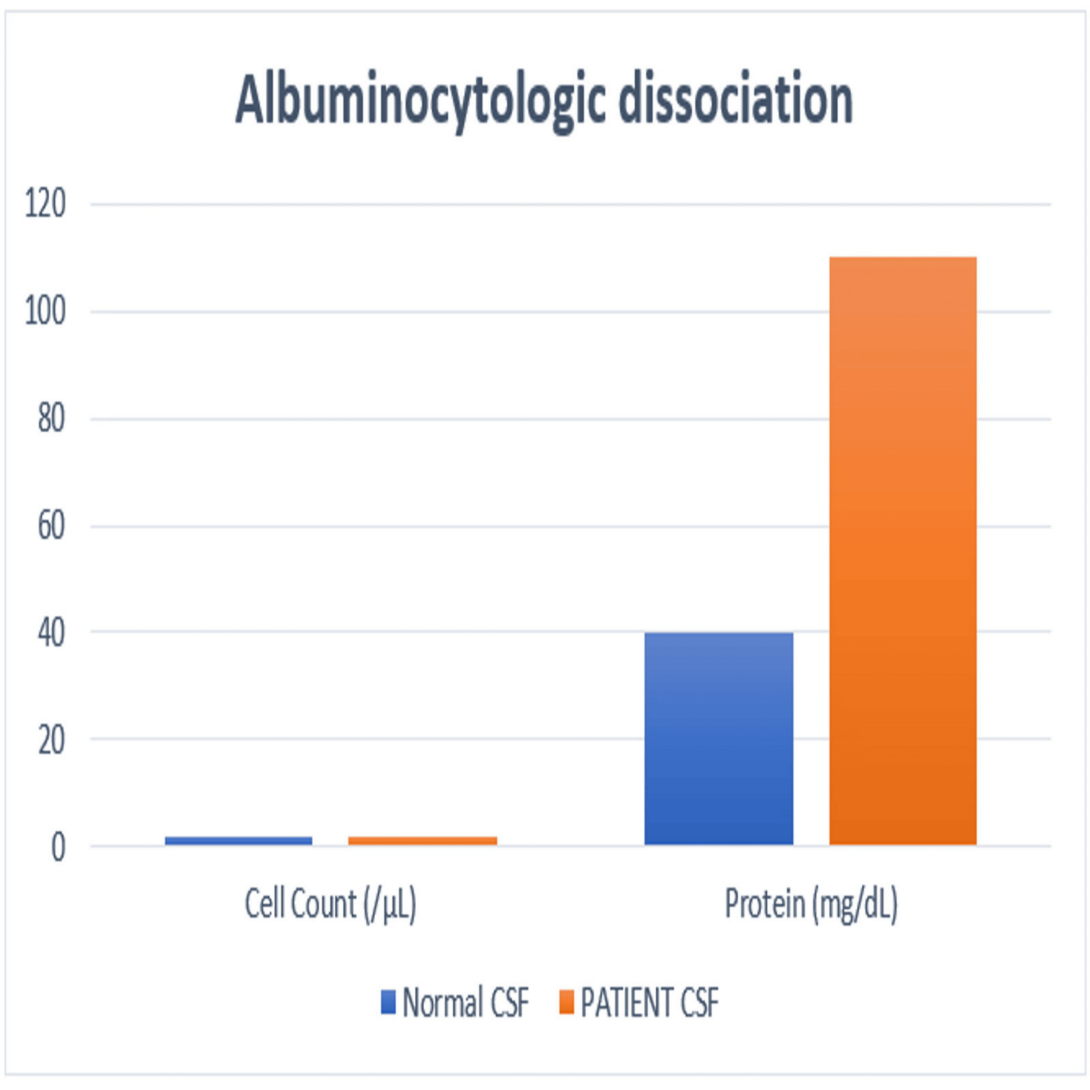
Cerebrospinal fluid profile comparing normal values with findings in the patient, demonstrating albuminocytologic dissociation.

Routine laboratory tests, including hematological counts, renal and liver function tests, and electrolytes, were normal. The diphtheria throat swab was negative. Cerebrospinal fluid analysis showed a protein level of 110 mg/dL (normal 15-60 mg/dL) with 2 cells/mm³, consistent with albuminocytologic dissociation (Figure 2). Brain and cervical spine MRI findings were normal, with no evidence of infarction or demyelination. Nerve conduction studies (NCS) demonstrated prolonged distal latencies, reduced compound muscle action potential (CMAP) amplitudes, and absent F-waves in the upper limbs, indicating a demyelinating motor polyneuropathy predominantly affecting the upper limbs. Serology revealed positive anti-GQ1b IgG and anti-GT1a IgG antibodies. Antibodies to acetylcholine receptors and MuSK receptors were negative (Table 1).

**Table 1:** Serological test results showing key antibody findings in the patient.

Test name	Result
Anti-ganglioside antibodies IgG	
GM1	Negative
GM2	Negative
GM3	Negative
GD1a	Negative
GD1b	Negative
GT1a	Positive
GT1b	Negative
GQ1b	Positive
Antibodies against acetylcholine receptor	Negative
Antibodies against MuSK receptors	Negative

A diagnosis of GBS with overlapping MFS and PCB variants was established based on the clinical presentation, serological findings, and electrophysiological results. The patient was treated with intravenous immunoglobulin (IVIG) at 0.4 g/kg/day for five days, along with supportive care that included nasogastric feeding due to aspiration risk, close respiratory monitoring (no ventilatory support was required), and initiation of physiotherapy.

By day 7, the patient showed gradual improvement in ptosis and neck muscle strength. By week 3, there was near-complete recovery of ophthalmoplegia and upper limb function. At the three-month follow-up, the patient had fully recovered, with normal neurological examination findings, negative repeat anti-GQ1b antibody testing, and normalized nerve conduction study results (Table 2).

**Table 2:** Timeline of events, from onset of symptoms to complete recovery.

Day 0	Fever episode
Day 1-11	Progressive symptoms of diplopia, drooping of eye lids, dysarthria, and dysphagia, accompanied by neck weakness and upper limbs weakness
Day 12	Presentation in emergency department
Day 13-19	Progressive worsening
Day 15	Starting of IVIG
Day 20	Mild improvement
Week 4	Near-complete recovery
3 months	Full recovery with normal tests

## Discussion

GBS encompasses a broad group of acute immune-mediated polyradiculoneuropathies. The classic form is defined by ascending symmetrical weakness and areflexia, while atypical variants such as MFS and PCB present with more localized or distinct clinical features. This case, showing characteristics of both MFS and PCB variants, adds to the growing evidence that GBS subtypes fall along a continuum rather than functioning as isolated clinical entities [3].

The overlap of MFS and PCB is exceptionally rare but provides important insight into the clinical-immunological spectrum of GBS. One well-documented report described reversible nodo-paranodal conduction failure in an MFS/PCB overlap, supported by serial nerve conduction studies that showed rapid normalization of motor responses and delayed recovery of sensory potentials, findings consistent with reversible conduction failure rather than demyelination or axonal loss [6].

Another case described a 55-year-old man who was initially misdiagnosed with a possible posterior circulation stroke. He developed ophthalmoplegia, ataxia, mild dysarthria, and bilateral sensory loss in the hands, followed later by bulbar symptoms. Standard CT and MRI were normal, and once the overlap syndrome was recognized and IVIG was started, he showed rapid and sustained recovery [7]. Such cases highlight the need to maintain a broad differential diagnosis in patients presenting with cranial or bulbar deficits.

Our case aligns with this existing literature while contributing additional value by demonstrating dual antibody positivity (anti-GQ1b and anti-GT1a) along with an electrophysiological pattern of demyelinating motor neuropathy that predominantly affected the upper limbs. This antibody profile supports the diagnosis and helps distinguish these variants from mimics such as brainstem stroke or myasthenia gravis, where serology and conduction patterns differ. Together, the clinical picture, antibody findings, and neurophysiology provided a strong diagnostic foundation.

The GBS spectrum model proposed by Wakerley and Yuki conceptualizes these overlaps as part of a broader immunological and clinical continuum rather than discrete syndromes [4]. In one study of 60 patients with Fisher Syndrome, 50% demonstrated overlap features: 23% had PCB-type involvement, 15% progressed to classic GBS, and 12% showed characteristics of Bickerstaff brainstem encephalitis. These findings emphasize the dynamic and overlapping nature of these disorders [8].

From a prognostic standpoint, early initiation of IVIG is key. Previous reports associate timely IVIG therapy with marked improvement and full recovery over weeks to months. Our patient achieved complete recovery within three months, reinforcing the benefit of early immunotherapy in atypical or overlapping GBS presentations [7].

## Conclusions

This example underscores the necessity for increased clinical vigilance about unusual and overlapping manifestations of GBS, especially in resource-limited environments where ganglioside antibody testing may be unavailable. Misdiagnosis or delayed intervention can result in considerable morbidity, including respiratory failure or progression of cranial nerve impairment. Consequently, doctors must uphold a heightened level of skepticism when faced with cranial nerve involvement accompanied by areflexia, even in the absence of typical ascending paralysis. By prioritizing accurate diagnosis and timely treatment, healthcare providers can mitigate the risks associated with GBS. This approach not only improves patient outcomes but also increases awareness about the disease's diverse presentations.
